# High Visceral Fat Area Attenuated the Negative Association between High Body Mass Index and Sarcopenia in Community-Dwelling Older Chinese People

**DOI:** 10.3390/healthcare8040479

**Published:** 2020-11-12

**Authors:** Cheng Li, Bingxian Kang, Ting Zhang, Hongru Gu, Qingqing Man, Pengkun Song, Zhen Liu, Jingyi Chen, Xile Wang, Bin Xu, Wenhua Zhao, Jian Zhang

**Affiliations:** 1 National Institute for Nutrition and Health, Chinese Center for Disease Control and Prevention, 27 Nanwei Road, Xicheng District, Beijing 100050, China; lichengyys@126.com (C.L.); qqm0327@163.com (Q.M.); songpk@ninh.chinacdc.cn (P.S.); liuzhen@ninh.chinacdc.cn (Z.L.); jingyich@126.com (J.C.); zhaowh@chinacdc.cn (W.Z.); 2 Wuyuan County Center for Disease Control and Prevention, 105 Shiji Road, Wuyuan, Inner Mongolia 015100, China; kbingxian@163.com (B.K.); wyxjkzxwxl@126.com (X.W.); 3 Yuexiu District Center for Disease Control and Prevention, 23 Jiaochang West Road, Guangzhou 510030, China; tingzhangyx@yeah.net (T.Z.); xubin6710@163.com (B.X.); 4 Taicang City Center for Disease Control and Prevention, 36 Xianfu West Street, Taicang 215400, China; bcghr@126.com

**Keywords:** sarcopenia, body mass index, visceral fat area, obesity, community-dwelling older people

## Abstract

The association between obesity and sarcopenia remains controversial. The present study was conducted to explore the associations among body mass index (BMI), visceral fat area (VFA), and sarcopenia in older people and analyze their potential mechanisms. This cross-sectional study included 861 community-dwelling older Chinese people from three regions of China. BMI, VFA, muscle mass, muscle strength, physical performance, body composition, and metabolic markers were measured. Muscle mass and muscle strength were positively correlated with BMI, but were negatively correlated with VFA. Simple overweight/obesity was negatively associated with sarcopenia (OR = 0.07, 95% CI = 0.03~0.18), and the OR value was lower than combined obesity (OR = 0.16, 95% CI = 0.09~0.28). Fat free mass and total body protein were positively associated with high BMI but negatively associated with high VFA. Furthermore, high VFA was adversely associated with some metabolic risk factors of sarcopenia. Combination of BMI and VFA increased diagnostic efficiency of low muscle mass and sarcopenia. In conclusion, high BMI was negatively associated with sarcopenia, while high VFA attenuated the negative association between high BMI and sarcopenia. The opposite association may partially be attributed to their different associations with body composition and metabolic risk factors of sarcopenia. Therefore, bedsides BMI, VFA and its interaction with BMI should be considered in sarcopenia prevention.

## 1. Introduction

Sarcopenia has been characterized as age-related low muscle mass (LMM), combined with the decline of muscle strength and physical performance [1,2]. Sarcopenia, which is closely associated with disability, poor quality of life, and increased mortality in older people, has gradually attracted considerable attention around the world [1,2,3,4].

Previous studies indicated that, muscle tissue dysfunction and muscle fiber insufficiency, as the main outcomes of aging-related obesity, may lead to muscle recession and sarcopenia [5,6]. As the most commonly used indicator of obesity, body mass index (BMI) has been widely used to detect the association between obesity and sarcopenia [6,7]. However, several previous studies reported negative associations between overweight/obesity defined by high BMI and sarcopenia [8,9]. A cross-sectional study in Austria found that, compared with their normal weight counterparts, overweight women had a significantly negative association with sarcopenia [8]. A cohort study in China found high BMI was protective against sarcopenia and increased its reversibility in older people after a four-year follow-up [9]. Due to the limitation of BMI in differentiating between lean body mass and body fat tissue, some researchers considered that BMI may not be a proper index to discuss the association between obesity and sarcopenia [10].

Compared with BMI-defined obesity, body fat accumulation during age-related obesity in older people has been considered as a more important risk factor of sarcopenia in older people [5,10,11]. Intermuscular adipocytes hypertrophy and intramyocellular lipid overaccumulation caused by high body fat mass could provoke dysfunction in skeletal muscle cells and inhibit muscle protein synthesis [12,13,14]. Meanwhile, visceral fat increase could induce systematic inflammation and insulin resistance [15,16], which in turn could cause LMM and sarcopenia in older people [17,18]. Those results indicated that body fat overaccumulation in different tissues may induce muscle decline and sarcopenia through multiple pathways.

A recent study in Japan found diabetes patients with high body fat mass and low BMI may be more likely to develop sarcopenia [19]. Meanwhile, during the normal progression of obesity, the increasement of lean mass is generally accompanied be a larger increase in fat mass [20]. This phenomenon suggested that, besides the particular effect of BMI on sarcopenia or the particular effect of body fat mass on sarcopenia, the interaction between BMI and body fat mass should also be considered in the development of sarcopenia. Furthermore, several recent studies indicated that, compared with subcutaneous fat, visceral fat showed more intense association with metabolic dysfunction in older people [21,22]. Visceral fat directly is linked to inflammation, liver dysfunction, and insulin resistance, which have been associated with muscle decline and sarcopenia in older people in previous studies [23,24]. Several metabolic markers related to inflammation and liver function, such as high sensitivity C-reactive protein (hsCRP), γ-glutamyl transpeptidase (GGT) and alanine aminotransferase (ALT), have been associated with sarcopenia in older people in previous studies [18,25,26]. Those results indicated visceral fat may be a more sensitive predictor of muscle recession and sarcopenia than total body fat in older people. Nevertheless, the interaction between BMI and visceral fat on sarcopenia and the potential mechanisms are still unclear [23,27,28].

Therefore, the present cross-sectional study evaluated the associations of BMI, visceral fat area (VFA), and their interactions with sarcopenia and its components in community-dwelling older Chinese. Relevant mechanisms affecting muscle health, such as body composition and metabolic risk-factors of sarcopenia, were also assessed to explore the potential mechanisms related to the different associations among BMI, VFA, and sarcopenia. Furthermore, we developed screening models for LMM and sarcopenia based on the combination of BMI and VFA in community-dwelling older people.

## 2. Materials and Methods

### 2.1. Study Design and Population

**Figure 1:**
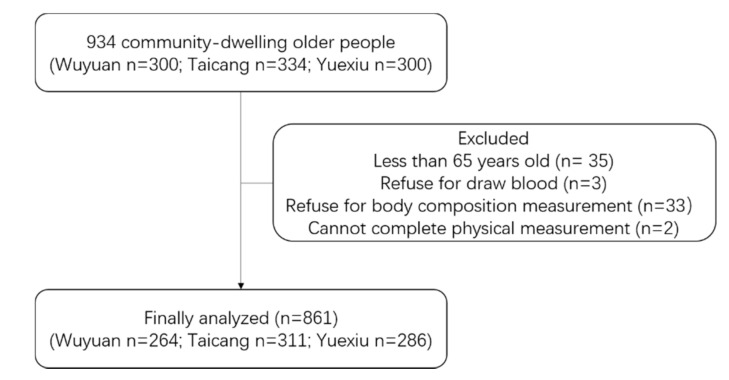
Flow diagram.

In 2018, community-dwelling older people were recruited from three regions in China, Wuyuan in the north of China, Taicang in the east of China, and Yuexiu in the south of China. In each region, about 300 older people were recruited from 2 to 4 randomly selected communities. Flow diagram was shown in Figure 1. Individuals with the following conditions were excluded: (1) under 65 years old; (2) disability for daily activity; (3) pacemaker or severe edema; (4) obstacle for communication; (5) refusal of blood draws. All the participants signed an informed consent before enrollment. A total of 934 community-dwelling older participants were recruited, and 861 participants were finally analyzed in the present study. This study was approved by the Ethics Committee of the National Institute of Nutrition and Health of Chinese Center for Disease Control and Prevention, in accordance with the Helsinki Declaration (2018-014).

### 2.2. Anthropometric Measurements

All the anthropometric measurements were conducted by trained doctors and nurses in local community hospitals. BMI (kg/m^2^) was calculated as weight divided by the square of body height. To guarantee the accuracy and feasibility of body composition measurement in different regions, skeletal muscle mass, VFA, body composition, such as fat free mass (FFM), body fat percentage (PBF), total body protein, and total body minerals, were measured by the bioelectrical impedance analysis device (InBody 770, Biospace, Seoul, Korea) [2,29]. Skeletal muscle mass index (SMI, kg/m^2^) was calculated as appendicular skeletal muscle mass (ASM) divided by the square of height. Handgrip strength was tested twice for both the dominant and non-dominant hand, using a handheld dynamometer (CAMRY EH101, Xiangshan, Guangdong, China) [30]. Maximum data were recorded as handgrip strength (kg). All the participants were asked to walk twice in a 4-m course at their usual walking speed. Gait speed (m/s) was then calculated by 4-m length divided by the average time.

### 2.3. Laboratory Measurements

All blood samples were obtained in the morning after a 12-h overnight fast. Serum samples were stored in −80 °C after centrifugation (15 min, 3000 rpm). Metabolic markers related to muscle health and sarcopenia, such as alkaline phosphatase (ALP), GGT, ALT, aspartate aminotransferase (AST), cholinesterase (ChE), and hsCRP, were measured by an automatic biochemical analyzer (Hitachi 7600, Hitachi, Tokyo, Japan).

### 2.4. Obesity and Sarcopenia

Obesity and classification: (1) Individuals with BMI < 18.5 kg/m^2^ were identified as underweight; 18.5 ≤ BMI < 24 kg/m^2^ were identified as normal weight; 24 ≤ BMI < 28 kg/m^2^ were identified as overweight; and BMI ≥ 28 kg/m^2^ were identified as obesity, according to standard of China, which was recommend by the Working Group on Obesity in China, and widely used in large-scale epidemic surveys in China [31,32,33,34,35]. BMI ≥ 24 kg/m^2^ were identified as high BMI in the present study. (2) Individuals with VFA ≥100 cm^2^ were identified as visceral obesity [36]. VFA ≥ 100 cm^2^ were identified as high VFA in the present study. (3) Individuals with BMI < 24 kg/m^2^ and VFA < 100 cm^2^ were classified into non-obesity group (NOB); BMI < 24 kg/m^2^ and VFA ≥ 100 cm^2^ were classified into visceral obesity group (VOB); BMI ≥ 24 kg/m^2^ and VFA < 100 cm^2^ were classified into simple overweight/obesity group (SOB); BMI ≥ 24 kg/m^2^ and VFA ≥ 100 cm^2^ were classified into combined obesity group (COB).

Sarcopenia: (1) Male participants with handgrip strength < 28 kg (female < 18 kg) were identified as low handgrip strength. (2) Participants with gait speed < 1.0 m/s were identified as low gait speed. (3) Male participants with SMI < 7 kg/m^2^ (female < 5.7 kg/m^2^) were identified as LMM. (4) Participants with LMM and low handgrip strength or low gait speed were identified as sarcopenia according to the criteria of Asian Working Group for Sarcopenia (AWGS) updated in 2019 [37].

### 2.5. Covariates

Relevant data were collected through a face-to-face interview. Collected information was related to socio-demographics for area, age, gender, and education level; lifestyles for living condition and physical activity; diagnosis history of cardiovascular disease and type 2 diabetes.

### 2.6. Statistical Analysis

Data are expressed as mean ± standard deviation (SD) or percentage. Categorical variables were compared by chi-squared tests. Pearson or Spearman correlations were performed according to the distribution of data (normal distribution or otherwise). Partial correlations were performed with the adjustment of relevant covariates, and BMI or VFA. Associations among high BMI, high VFA and sarcopenia were analyzed by multiple logistic regression analyses, with and without the adjustment of relevant covariates, including age, gender, region, education level, living condition, physical activity, and non-communicable diseases. Odds ratio (OR) and 95% confidence interval (CI) were obtained in logistic regression model. General linear models (GLMs) were performed to evaluate the associations of high BMI and high VFA and their interactions with body composition and relevant metabolic risk factors, with the adjustment of relevant covariates. If continuous variables did not fit normal distribution, Box-Cox power transformations were performed to normalize the data in GLMs. Receiver operating characteristic (ROC) curves were performed to evaluate and compare the diagnostic efficiency of BMI, VFA and their combination on LMM and sarcopenia. All data analyses were performed using statistical analysis system (SAS), (V9.4, SAS Institute, Cary, NC, USA). *p* < 0.05 was considered statistically significant.

## 3. Results

### 3.1. Participants Characteristics

Demographic characteristics of subjects with and without sarcopenia were shown in Table 1. A total of 861 community-dwelling older subjects were analyzed in the study. Prevalence rate of sarcopenia was 19.9% (171/861), according to the criteria of AWGS updated in 2019. Lower prevalence rate of sarcopenia was found in younger subgroup (14.5%, 98/676), overweight subgroup (6.5%, 20/306), obesity subgroup (6.1%, 6/99) and visceral obesity subgroup (13.4%, 42/314).

### 3.2. Correlations among BMI, VFA and Muscle Health Indicators

Correlations among BMI, VFA, and muscle health indicators were presented in Table 2. BMI was positively correlated with handgrip strength (*r* = 0.10, *p* = 0.003), and SMI (*r* = 0.50, *p* < 0.001) without adjustment. The coefficients were increased in partial correlation models for handgrip strength (*r* = 0.14, *p* < 0.001) and SMI (*r* = 0.70, *p* < 0.001), with the adjustment of age, gender, area, education level, living condition, physical activity, non-communicable diseases, and VFA. VFA was negatively correlated with handgrip strength (*r* = −0.12, *p* = 0.003) and gait speed (*r* = −0.12, *p* = 0.004) without adjustment. However, the negative correlation between VFA and handgrip strength (*r* = −0.07, *p* = 0.040) decreased in the partial correlation models with the adjustment of covariates and BMI. Similar changes were also observed in the correlation between VFA and gait speed (*r* = −0.07, *p* = 0.034). Furthermore, VFA was moderately and positively associated with SMI (*r* = 0.11, *p* = 0.002) in the correlation model without adjustment. After the adjustment of covariates and BMI, VFA showed a strongly negative association with SMI (*r* = −0.44, *p* < 0.001).

### 3.3. Associations among High BMI, High VFA, and Sarcopenia

Multiple logistic regressions were performed to explore the associations among high BMI, high VFA, and sarcopenia (Table 3). Compared with the NOB group, subjects in the COB group were negatively associated with low handgrip strength in crude model (OR = 0.71, 95% CI = 0.50~0.99). Low gait speed was positively associated with COB (OR = 1.85, 95% CI = 1.35~2.54) in the crude model, but both the significant associations were attenuated and disappeared after the adjustment of age, gender, area, education level, living condition, physical activity, and non-communicable diseases. Compared with NOB, SOB and COB showed strongly negative associations with LMM, while the ORs of COB for LMM in the crude models and adjusted models were both much higher than SOB (0.12 vs. 0.04, 0.10 vs. 0.03), indicating a weaker negative association of COB than SOB with LMM. In terms of sarcopenia, significantly positive association was observed in VOB in the crude model (OR = 2.23, 95% CI = 1.18~4.19), but the positive association disappeared in the adjusted model (OR = 1.90, 95% CI = 0.93~3.86). Compared with NOB, SOB and COB both were negatively associated with sarcopenia, while the ORs of COB in crude models and adjusted models were both much higher than ORs of SOB (0.20 vs. 0.09, 0.16 vs. 0.07), which indicates a weaker negative association of COB than SOB with sarcopenia.

### 3.4. Associations among High BMI, High VFA, and Body Composition

Associations among high BMI, high VFA, and body composition were analyzed in GLMs with the adjustment of relevant covariates (Table 4). For body composition, FFM was positively associated with high BMI (*p* < 0.001) and was negatively associated with high VFA (*p* = 0.018). The highest FFM level was found in the SOB group (46.57 ± 6.59 kg) and the lowest level was found in the VOB group (34.25 ± 4.84 kg). ASM was also positively associated with high BMI (*p* < 0.001), and the highest level of ASM was found in the SOB group (19.30 ± 3.43 kg). PBF was, respectively, associated with high BMI (*p* < 0.001) and high VFA (*p* < 0.001). The level of PBF in COB (38.59 ± 5.27%) was significantly higher than VOB (37.33 ± 2.68%) and SOB (28.39 ± 3.85%). Significantly positive association was found between total body protein and high BMI (*p* < 0.001), while a negative association was found between total body protein and high VFA (*p* = 0.015). The highest level of total body protein was found in the SOB group (9.08 ± 1.32 kg), and the lowest level was found in the VOB group (6.60 ± 0.96 kg). Similar opposite influences of high BMI and high VFA were also observed in their association with total body minerals.

### 3.5. Associations among High BMI, High VFA, and Metabolic Risk Factors of Sarcopenia

For the metabolic risk factors of sarcopenia, high VFA showed the adverse associations with liver metabolism and inflammation in the present study (Table 5). In the adjusted GLMs, high VFA showed significantly positive association with serum GGT (*p* = 0.005) and ChE (*p* = 0.008) and showed a negative association with ALT (*p* = 0.049), which may lead to liver dysfunction in older people. The ratio of AST/ALT was negatively associated with high BMI (*p* = 0.008) and high VFA (*p* = 0.006), respectively. The lowest ratio of AST/ALT was observed in the SOB group (1.57 ± 0.73). HsCRP was also significantly associated with high VFA (*p* < 0.001). The highest level of hsCRP was found in COB group (3.33 ± 4.46 mg/L), while the lowest level was found in the SOB group (1.86 ± 3.39 mg/L).

### 3.6. Combination of BMI and VFA on the Prediction of LMM and Sarcopenia

**Figure 2:**
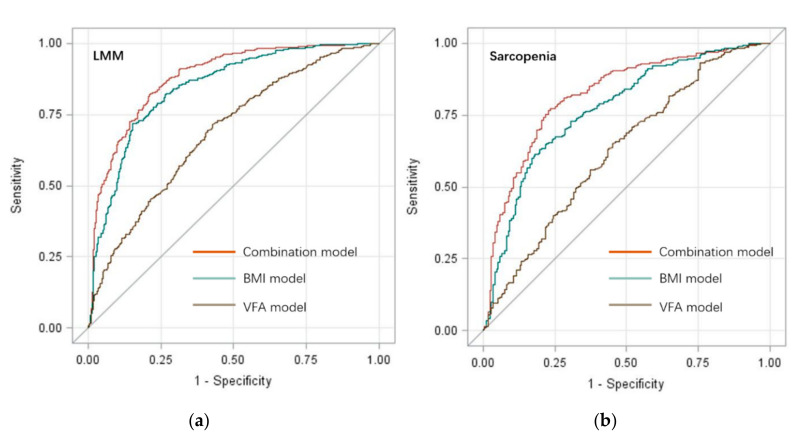
Receiver operating characteristic curves of BMI, VFA, and their combination to predict LMM and sarcopenia in older Chinese people. (**a**) Predict models for LMM; (**b**) Predict models for sarcopenia.

The intense but opposite correlations among BMI, VFA, and sarcopenia suggested the potential meaning of BMI and VFA in the screening for sarcopenia. ROC curves were performed to assess the diagnostic efficacy of BMI, VFA, and their combination on LMM and sarcopenia (Figure 2), respectively. Area under the curve (AUC) value of the combined screening model for LMM was 0.88 (95% CI = 0.86~0.90), and was significantly higher than BMI model (AUC = 0.84, 95% CI = 0.82~0.87, *p* < 0.001) and VFA model (AUC = 0.69, 95% CI = 0.65~0.73, *p* < 0.001). AUC value of the combined screening model for sarcopenia was 0.82 (95% CI = 0.78~0.86) and was significantly higher than the BMI (AUC = 0.77, 95% CI = 0.73~0.81, *p* < 0.001) model and VFA model (AUC = 0.63, 95% CI = 0.58~0.67, *p* < 0.001). The combination of BMI and VFA showed high diagnostic efficacy on LMM and sarcopenia in older people. Based on the combination of BMI and VFA, simple-to-use nomograms were performed for the screening of LMM and sarcopenia (Figures S1 and S2).

## 4. Discussion

Previous studies indicated that underweight older subjects with low BMI were more susceptible to LMM and sarcopenia [38]. Without the consideration of body fat, older subjects with high BMI tend to have a larger amount of lean body mass, and then they tend to have a sufficient quantity of muscle mass and low risk of LMM [23]. Meanwhile, increasing accumulation of body fat, especially visceral fat, could increase the risk of muscle protein wasting, systematic inflammation, and insulin resistance, then increasing the risk of muscle mass decline and sarcopenia in older people [23,24]. A previous cohort study in Australia found that the increase of body fat mass accompanied with the increase of BMI and the decline of lean body mass, could increase the risk of sarcopenia and fragility in adult males [39]. Due to the limitation of separating lean body mass and fat mass, BMI could not evaluate the changes in body composition during weight gain [28]. Therefore, it is necessary to evaluate the relationship between BMI and body fat, especially visceral fat, in the progression of muscle mass decline and sarcopenia.

In the present study, the association of BMI and VFA with sarcopenia and its components was assessed in community-dwelling older people in China. BMI was positively correlated with muscle strength and muscle mass, while VFA showed significantly negative correlations with them. High BMI without visceral obesity was negatively associated with LMM and sarcopenia. While high VFA attenuated the negative association between high BMI and LMM and sarcopenia. Further analysis indicated that the opposite associations of BMI and VFA with LMM and sarcopenia may be partially attributable to their different associations with body composition and metabolic risk factors of sarcopenia. Due to the significant but opposite associations of BMI, VFA with LMM, and sarcopenia, we developed screening tools for LMM and sarcopenia, based on the combination of BMI and VFA for the first time. Our results provided scientific evidence for the diagnosis and prevention of sarcopenia in community-dwelling older people.

As a surrogate of muscle strength, handgrip strength has been measured, and its association with BMI and VFA has been discussed in the present study. A weak positive correlation between handgrip strength and BMI has been shown in Table 2 (*r* = 0.14, *p* < 0.001). Meanwhile, the correlation coefficient between handgrip strength and BMI increased after the adjustment of VFA and relevant covariates, this change may partially attribute to the negative correlation between VFA and handgrip strength. The increase of body fat during bodyweight gain may increase the risk of inflammation, insulin resistance, and muscle cell atrophy, and lead to the decline of muscle mass and strength [12,13,17]. A significantly negative association was observed between low handgrip strength and COB, while no significant association was observed between low handgrip strength and other types of obesity in the present study. Meanwhile, the positive association between COB and low handgrip strength were attenuated and disappeared after adjustment. This change reflected the complexity of handgrip strength decline in older subjects. During the aging progress, handgrip strength could be affected by many influencing factors. In addition to BMI and body fat, physical activity, living condition, blood pressure, smoking, stress, chronic disease, and renal function have been associated with handgrip strength in older subjects, and those factors seem to be more important than BMI in handgrip strength decline [40,41]. As a surrogate of physical performance, gait speed only showed a weak correlation with VFA. The positive association between COB and low gait speed was also attenuated and disappeared in the adjusted logistic regression model. This change may be partially attributed to the weak correlation between VFA and gait speed, and the numerous influencing factors of gait speed decline in older people, such as knee osteoarthritis, dementia, and balance disorder [29,42].

Different with handgrip strength and gait speed, SMI showed a strong correlation with BMI (*r* = 0.70, *p* < 0.001) in the present study. Compared with the NOB group, SOB was negatively associated with LMM (OR = 0.03, 95% CI = 0.01~0.07) and sarcopenia (OR = 0.07, 95% CI = 0.03~0.18), indicating that high BMI was negatively associated with sarcopenia in older subjects. High BMI showed no significant associations with low handgrip strength and low gait speed, therefore, the negative association between high BMI and sarcopenia mainly attributed to its significantly negative association with LMM. Meanwhile, COB was negatively associated with sarcopenia, while the OR value of sarcopenia in COB group were much higher than SOB group (0.16 vs. 0.07), indicating the negative association between high BMI and sarcopenia could be attenuated by high VFA. Though high VFA could not reverse the negative association between high BMI and sarcopenia, it could increase the risk of muscle protein wasting, systematic inflammation and insulin resistance, then increased the risk of muscle mass decline and sarcopenia in subjects with normal BMI [6,23,43]. Therefore, it is necessary to evaluate the relationship and explore the interplay between BMI and visceral fat in the development of sarcopenia.

The opposite association of BMI and VFA with sarcopenia may be partially mediated by their different associations with body composition. Lean body mass and fat mass both increased with weight gain [20], and a positive correlation was observed between BMI and body fat mass in older people [9], suggesting BMI and VFA may have different associations with body composition. Nevertheless, their interactions on body compositions remain unclear. In the present study, high VFA was negatively associated with FFM and ASM, and was positively associated with PBF. Meanwhile, high BMI was positively associated with FFM and ASM, and was negatively associated with PBF. Further analyses indicated that the highest level of total body protein was found in the SOB group, while the lowest level was found in the VOB group. The negative association between total body protein and visceral obesity may be because of the insulin resistance caused by high VFA [16]. Insulin stimulated muscle protein synthesis by transporting circulating amino acids into skeletal muscle cells [44]. Meanwhile, insulin also played a vital role in muscle protein breakdown [45]. The role of insulin in the regulation of protein metabolism indicated the adverse effects of insulin resistance and high VFA on muscle protein metabolism in older subjects. In addition to total body protein, similar negative association was also observed between total body minerals and VOB. It should be noticed that, though high VFA showed a significantly adverse association with body composition, it could not reverse the negative association of high BMI with LMM and sarcopenia in the present study. The negative association between high BMI and sarcopenia was much stronger than the positive association between high VFA and sarcopenia, which may be caused by the intense association between SMI and BMI.

In addition to body composition, metabolic dysfunction and systematic inflammation have been proven to be involved in the development of muscle decline and sarcopenia [46]. Some metabolic markers, such as GGT, ALT, and hsCRP, have been identified as metabolic predictors of sarcopenia in older people [18,25,26]. Evaluated GGT, positively associated with non-alcoholic fatty liver disease, adiposity, and insulin resistance, has been proven to be an independent risk factor of sarcopenia in community-dwelling older subjects [25]. Partially in line with the previous study, the highest level of GGT was found in the COB group in the present study, which indicated a higher risk of sarcopenia in older subjects. In previous studies, ALT level was consistently associated with BMI, and subjects with lower BMI and lower ALT tended to be a worse nutritional status [26,47]. Inversely, elevated ALT, commonly observed in patients with fatty liver disease, may increase the risk of metabolic dysfunction and insulin resistance [47,48,49]. Meanwhile, a previous study found low ALT level was a predictor for pyridoxine deficiency, frailty, and sarcopenia in older people, which may be associated with liver impairment and malnutrition [26]. Since a low AST/ALT ratio has been associated with fatty liver disease in previous studies [50,51], the AST/ALT ratio was also analyzed in the present study, to further discuss the association between BMI, VFA, and sarcopenia. The levels of the AST/ALT ratio in the SOB group and COB group were significantly lower than the NOB group, indicating that both the high BMI and high VFA, with the highest risk of fatty liver disease, may increase the risk of sarcopenia. Different with the AST/ALT ratio, the significant change of hsCRP was only found in the COB group with high BMI and high VFA, and high VFA showed a positive association with serum hsCRP in GLM analysis. This result indicated that, compared with high BMI, high VFA may be a more dangerous indicator for metabolic dysfunction. High VFA could increase the risk of sarcopenia by triggering liver dysfunction and metabolic dysfunction.

In the present study, due to the limitation of BMI on the evaluation of body composition, and the strongly positive association between high VFA and sarcopenia, the combination of BMI and VFA was used to develop screening tools for LMM and sarcopenia. Sarcopenia diagnosis generally requires measurements of ASM, muscle strength, and physical performance in older people [29,37]. However, it is inconvenient to conduct generally screening for LMM and sarcopenia based on CT, MRI and dual-energy X-ray absorptiometry, because of the high equipment costs and the requirement for trained person to use the equipment [29,52]. Moreover, dementia, gait disorder, and hand disability in older people may also affect the measurements of muscle strength and physical performance [29]. Because of those limitations, some researchers developed some simple-to-use screening tools for LMM and sarcopenia via BMI, CC, and other anthropometric measurements [53,54,55]. Meanwhile, most of those screening tools, using a single anthropometric measurement, ignored the variation of body builds and nutrition status among individuals [53]. In the present study, based on the associations among high BMI, high VFA and sarcopenia, BMI, VFA, and their combination were used to develop screening tools for LMM and sarcopenia. Compared with single anthropometric measurement, the combination of BMI and VFA had significantly better diagnostic efficiency in the prediction of LMM (AUC = 0.88, 95% CI = 0.86~0.90) and sarcopenia (AUC = 0.82, 95% CI = 0.78~0.86). Those results indicated that besides BMI, VFA and their interactions should also be seriously considered in sarcopenia prevention and management. Furthermore, simple-to-use nomograms were developed for the identification of LMM (Supplementary Figure S1) and sarcopenia (Supplementary Figure S2) in older people in the present study.

The present study was not without any limitations. First, this study was a cross-sectional study, prospective cohort studies will be necessary for more powerful evidence in the future. Second, due to the absence of a uniform criteria of sarcopenia, criteria recommended by AWGS was used in this study. Furthermore, there are multiple types of obesity classification, based on body fat percentage, waistline and different cut-off values of BMI. Meanwhile, only two obesity classification standards were conducted in the present study. More kinds of obesity classification should be conducted in the future study to discuss the relationship between obesity and sarcopenia in older people.

## 5. Conclusions

High BMI was negatively associated with sarcopenia in community-dwelling older people, mainly attributable to its negative association with LMM. However, high VFA could attenuate the negative association between high BMI and sarcopenia. The opposite association of high BMI and high VFA with sarcopenia partially attributed to their different associations with body composition and relevant metabolic risk factors of sarcopenia. Combination of BMI and VFA significantly increased the diagnostic efficiency of LMM and sarcopenia. Therefore, bedsides BMI, VFA and its interaction with BMI should be considered in sarcopenia prevention.

## Figures and Tables

**Table 1:** Demographic characteristics.

Characteristic	Total	No Sarcopenia	Sarcopenia	*p*-Value
	*N* = 861	*N* = 690	*N* = 171	
**Age**				
65~74 year	676(78.5)	578 (85.5)	98 (14.5)	<0.001
75~ year	185(21.5)	112 (60.5)	73 (39.5)	
**Gender**				
Male	405 (47)	325 (80.3)	80 (19.8)	0.941
Female	456 (53)	365 (80)	91 (20)	
**Area**				
Yuexiu	286 (33.2)	232 (81.1)	54 (18.9)	0.694
Taicang	311 (36.1)	251 (80.7)	60 (19.3)	
Wuyuan	264 (30.7)	207 (78.4)	57 (21.6)	
**Education**				
Primary or below	531 (61.7)	416 (78.3)	115 (21.7)	0.245
Junior high school	176 (20.4)	146 (83)	30 (17)	
Senior high or above	154 (17.9)	128 (83.1)	26 (16.9)	
**Living condition**				
Living alone	87 (10.1)	68 (78.2)	19 (21.8)	0.130
Living with spouse	656 (76.2)	535 (81.6)	121 (18.5)	
Living with others	118 (13.7)	87 (73.7)	31 (26.3)	
**NCDs**				
Cardiovascular disease	178 (20.7)	144 (80.9)	34 (19.1)	0.776
Type 2 diabetes	113 (13.1)	96 (85)	17 (15)	0.169
**BMI**				
<18.5 kg/m^2^	49 (5.7)	17 (34.7)	32 (65.3)	<0.001
18.5~23.9 kg/m^2^	407 (47.3)	294 (72.2)	113 (27.8)	
24~27.9 kg/m^2^	306 (35.5)	286 (93.5)	20 (6.5)	
≥28 kg/m^2^	99 (11.5)	93 (93.9)	6 (6.1)	
**VFA**				
<100 cm^2^	547(63.5)	418 (76.4)	129 (23.6)	0.003
≥100 cm^2^	314(36.5)	272 (86.6)	42 (13.4)	

Data are presented as *n* (%). The percentages in “Total” were the column percentages within each subgroup. The percentages in “No sarcopenia” and “Sarcopenia” are row percentages. Categorical variables used chi-squared tests. NCDs, non-communicable diseases; BMI, body mass index; VFA, visceral fat area.

**Table 2:** Correlations among BMI, visceral fat area (VFA) and muscle health indicators.

Characteristic	BMI (kg/m^2^)				VFA (cm^2^)			
	*r* ^a^	*p*	*r* ^b^	*p*	*r* ^a^	*p*	*r* ^c^	*p*
Handgrip Strength(kg)	0.10	0.003	0.14	<0.001	−0.12	0.003	−0.07	0.040
Gait Speed(m/s)	−0.06	0.100	0.02	0.505	−0.12	0.004	−0.07	0.034
SMI (kg/m^2^)	0.50	<0.001	0.70	<0.001	0.11	0.002	−0.44	<0.001

^a^ Correlation coefficient. ^b^ Adjusted by age, gender, area, education level, living condition, physical activity, non-communicable diseases and VFA. ^c^ Adjusted by age, gender, area, education level, living condition, physical activity, non-communicable diseases and BMI. BMI, body mass index; VFA, visceral fat area; SMI, appendicular skeletal muscle index.

**Table 3:** Odds ratios (95% confidence intervals) for sarcopenia and its components according to high BMI and high VFA.

Characteristic	Model	NOB (N = 417)	VOB (N = 43)			*p*	SOB (N = 134)			*p*	COB (N = 271)			*p*
		Ref	OR	95% CI			OR	95% CI			OR	95% CI		
Low handgrip strength	Model 1	1	1.13	0.58	2.18	0.721	0.74	0.48	1.15	0.186	0.71	0.50	0.99	0.046
	Model 2	1	0.93	0.43	2.01	0.857	0.66	0.41	1.07	0.091	0.68	0.46	1.00	0.051
Low gait speed	Model 1	1	1.85	0.98	3.49	0.057	0.81	0.53	1.25	0.345	1.85	1.35	2.54	0.001
	Model 2	1	1.09	0.54	2.21	0.808	0.84	0.52	1.34	0.460	1.42	1.00	2.03	0.051
LMM	Model 1	1	1.71	0.87	3.38	0.120	0.04	0.02	0.09	<0.001	0.12	0.08	0.18	<0.001
	Model 2	1	1.57	0.75	3.28	0.234	0.03	0.01	0.07	<0.001	0.10	0.06	0.16	<0.001
Sarcopenia	Model 1	1	2.23	1.18	4.19	0.013	0.09	0.04	0.23	<0.001	0.20	0.12	0.32	<0.001
	Model 2	1	1.90	0.93	3.86	0.078	0.07	0.03	0.18	<0.001	0.16	0.09	0.28	<0.001

Data are presented as odds ratio (ORs) and 95% confidence intervals (CI). Model 1: unadjusted. Model 2: adjusted by age, gender, area, education level, living condition, physical activity and non-communicable diseases. NOB, non-obesity group; VOB, visceral obesity group; SOB, simple overweight and obesity group; COB, combined group; OR, odds ratio; CI, confidence interval.

**Table 4:** Associations among high BMI, high VFA, and body composition.

Characteristic	NOB (N = 413)	VOB (N = 43)	SOB (N = 134)	COB (N = 271)	*p* for Model	*p* for High VFA	*p* for High BMI	*p* for Interaction
FFM (kg)	39.88 ± 6.98	34.25 ± 4.84	46.57 ± 6.59 ^ab^	41.75 ± 7.47 ^ab^	<0.001	0.018	<0.001	0.833
ASM (kg)	16.06 ± 3.79	13.28 ± 2.72	19.30 ± 3.43 ^ab^	17.12 ± 3.97 ^ab^	<0.001	0.168	<0.001	0.767
PBF (%)	25.14 ± 6.01	37.33 ± 2.68 ^a^	28.39 ± 3.85 ^ab^	38.59 ± 5.27 ^abc^	<0.001	<0.001	<0.001	0.311
Body protein(kg)	7.74 ± 1.38	6.60 ± 0.96	9.08 ± 1.32 ^ab^	8.12 ± 1.47 ^ab^	<0.001	0.015	<0.001	0.784
Body minerals(kg)	2.71 ± 0.41	2.41 ± 0.29	3.10 ± 0.41 ^ab^	2.79 ± 0.47 ^abc^	<0.001	0.011	<0.001	0.513

Original data are presented as mean ± SD. *p*-values were obtained from GLMs, with the adjustment of age, gender, area, education level, living condition, physical activity and non-communicable diseases. If original variables did not fit normal distribution, Box-Cox power transformations were performed to normalize the data before analyses. ^a^ *p* < 0.05 vs. NOB; ^b^ *p* < 0.05 vs. VOB; ^c^ *p* < 0.05 vs. SOB. NOB, non-obesity group; VOB, visceral obesity group; SOB, simple overweight and obesity group; COB, combined obesity group; FFM, fat free mass; ASM, appendicular skeletal muscle mass; PBF, percent body fat mass.

**Table 5:** Associations among high BMI, high VFA, and metabolic risk factors of sarcopenia.

Characteristic	NOB (N = 413)	VOB (N = 43)	SOB (N = 134)	COB (N = 271)	*p* for Model	*p* for High VFA	*p* for High BMI	*p* for Interaction
ALP (U/L)	100.02 ± 32.34	101.91 ± 27.57	95.22 ± 25.78	101.98 ± 41.14	<0.001	0.562	0.487	0.712
GGT (U/L)	30.29 ± 32.71	35.93 ± 37.41	33.37 ± 27.93	38.35 ± 65.36 ^a^	<0.001	0.005	0.147	0.492
ALT (U/L)	15.17 ± 13.68	14.33 ± 5.84	17.67 ± 11.96	18.27 ± 15.83 ^a^	<0.001	0.049	0.066	0.567
AST (U/L)	23.50 ± 9.11	22.37 ± 5.54	23.53 ± 8.17	24.49 ± 14.77	<0.001	0.936	0.809	0.694
AST/ALT	1.83 ± 0.72	1.75 ± 0.67	1.57 ± 0.73 ^a^	1.64 ± 1.18 ^a^	<0.001	0.006	0.008	0.230
ChE (U/L)	366.72 ± 85.89	399.33 ± 98.61	370.57 ± 72.46	407.56 ± 74.25 ^a^	<0.001	0.008	0.175	0.902
hsCRP (mg/L)	2.22 ± 4.09	3.12 ± 4.88	1.86 ± 3.39	3.33 ± 4.46 ^ab^	<0.001	<0.001	0.257	0.912

Original data are presented as mean ± SD. *p*-values were obtained from GLMs, with the adjustment of age, gender, area, education level, living condition, physical activity and non-communicable diseases. If original variables did not fit normal distribution, Box-Cox power transformations were performed to normalize the data before analyses. ^a^ *p* < 0.05 vs. NOB; ^b^ *p* < 0.05 vs. VOB; NOB, non-obesity group; VOB, visceral obesity group; SOB, simple overweight and obesity group; COB, combined obesity group; ALP, alkaline phosphatase; GGT, γ-glutamyl transpeptidase; ALT, alanine aminotransferase; AST, aspartate aminotransferase; ChE, cholinesterase; hsCRP, high sensitivity C-reactive protein.

## Supplementary Materials

The following are available online at https://www.mdpi.com/2227-9032/8/4/479/s1, Figure S1: Regression nomogram for the screening of LMM in community-dwelling older people. Figure S2: Regression nomogram for the screening of sarcopenia in community-dwelling older people.

## Abbreviations

BMI	body mass index
VFA	visceral fat area
NOB	non-obesity group (BMI < 24 kg/m^2^, VFA < 100 cm^2^)
VOB	visceral obesity group (BMI < 24 kg/m^2^, VFA ≥ 100 cm^2^)
SOB	simple overweight and obesity group (BMI ≥ 24 kg/m^2^, VFA < 100 cm^2^)
COB	combined obesity group (BMI ≥ 24 kg/m^2^, VFA ≥ 100 cm^2^)
LMM	low muscle mass
SMI	skeletal muscle mass index
FMM	fat free mass
ASM	appendicular skeletal muscle mass
PBF	body fat percentage
ALP	alkaline phosphatase
GGT	γ-glutamyl transpeptidase
ALT	alanine aminotransferase
AST	aspartate aminotransferase
ChE	cholinesterase
hsCRP	high sensitivity C-reactive protein
AWGS	Asian Working Group for Sarcopenia
SD	standard deviation
OR	odds ratio
CI	confidence interval
GLM	general linear model
ROC	receiver operating characteristic curve
AUC	area under curve
