# Supplementary material for: High Visceral Fat Area Attenuated the Negative Association between High Body Mass Index and Sarcopenia in Community-Dwelling Older Chinese People
Source: Healthcare (Basel). 2020 Nov 12;8(4):479. doi: 10.3390/healthcare8040479 (PMC7712146; doi:10.3390/healthcare8040479)
Supplement: Supplementary file 1 [file healthcare-08-00479-s001.zip › healthcare-985014/healthcare-985014-Supplementary Figure S1.docx]

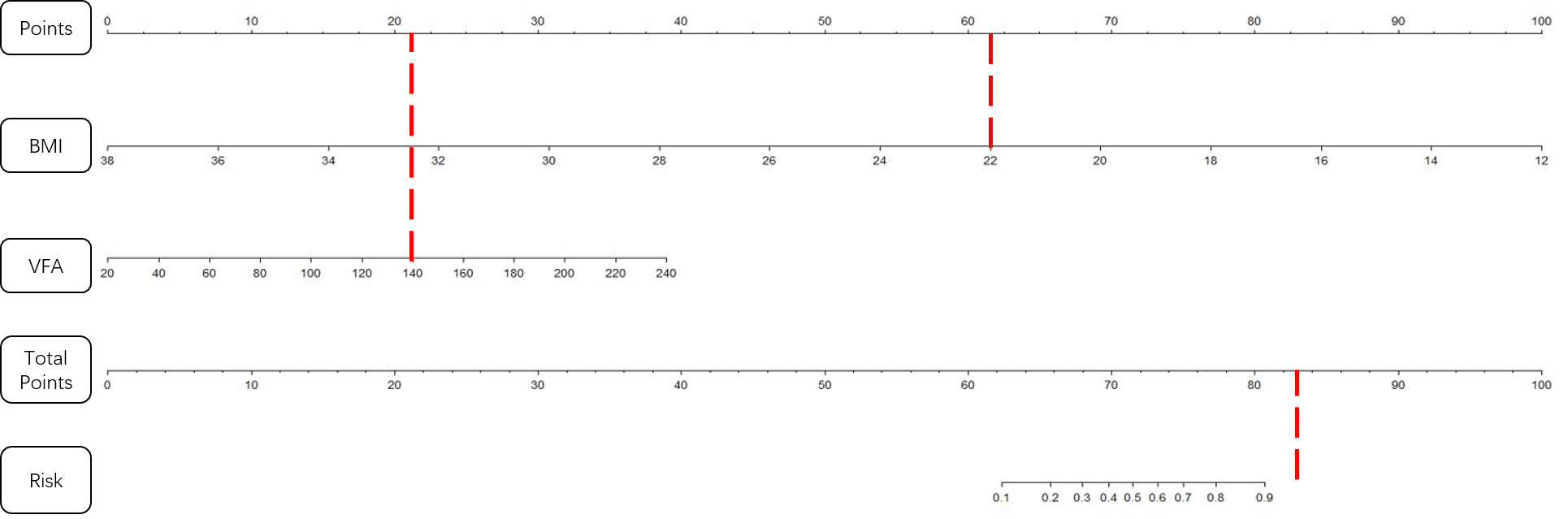


**Supplementary Figure S1.** Regression nomogram for the screening of LMM in community-dwelling older people.

Regression nomogram was drown for the prediction of LMM in Figure S1. The nomogram consisting of BMI and VFA, offered researchers a simple-to-use method for assessing LMM risk in community-dwelling older people. Total points were calculated by adding points from BMI and VFA in regression model. For example (red line), an older people, with BMI of 22 kg/m^2^ earned ~62 points, VFA of 140 cm^2^ earned ~21 points. Added together, this subject earned ~83 points, and the estimated probability of LMM was more than 90%.
